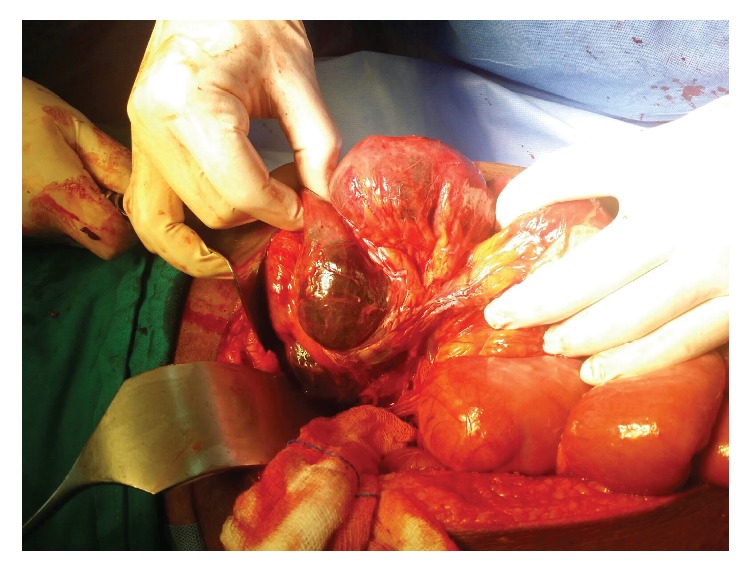# Intestinal Obstruction in a Patient with Sclerosing Encapsulating Peritonitis

**DOI:** 10.1155/2017/8316147

**Published:** 2017-03-26

**Authors:** Oday Obaid, Dawood Alhalabi, Mohamed Ghonami

**Affiliations:** Adan Hospital, Ahmadi, Kuwait

## Abstract

Sclerosing encapsulating peritonitis (SEP) is a rare disorder that is characterized by encapsulation of bowel loops by thick fibrinogenous case. Most patients present with vague abdominal symptoms. It is challenging to diagnose the condition preoperatively. Surgical management is preserved for patients with small bowel obstruction with no improvement on conservative measures or for those with signs of bowel ischemia (Li et al., 2014; Habib et al. 2011). Herein, we discuss the clinical signs and symptoms, the radiological features, the surgical management, and outcome of SEP based on a patient who underwent surgery in our hospital.

## 1. Introduction

Sclerosing encapsulating peritonitis, or cocoon syndrome, is a rare disorder, which is usually seen in patients on continuous ambulatory peritoneal dialysis (CAPD). The exact known of the condition is still unknown. The gross pathology resembles a cocoon case encapsulating the bowel loops, hence the name. Patients with SEP often present with vague abdominal symptoms. A computed tomography in a patient with intestinal obstruction is likely to raise the suspicion of SEP; however, the case is usually diagnosed intraoperatively. Many patients would resolve with only conservative medical managements, but some may require surgical intervention. It is still unclear whether early surgical intervention has an advantage over conservative management, but, in most reviewed case reports, surgeons preferred to preserve the surgical management for those who do not respond to conservative measures [1, 2]. Hereby, we discuss a case of SEP, according to a patient who presented to our clinic and underwent surgical intervention.

## 2. Case Presentation

**Figure 1:**
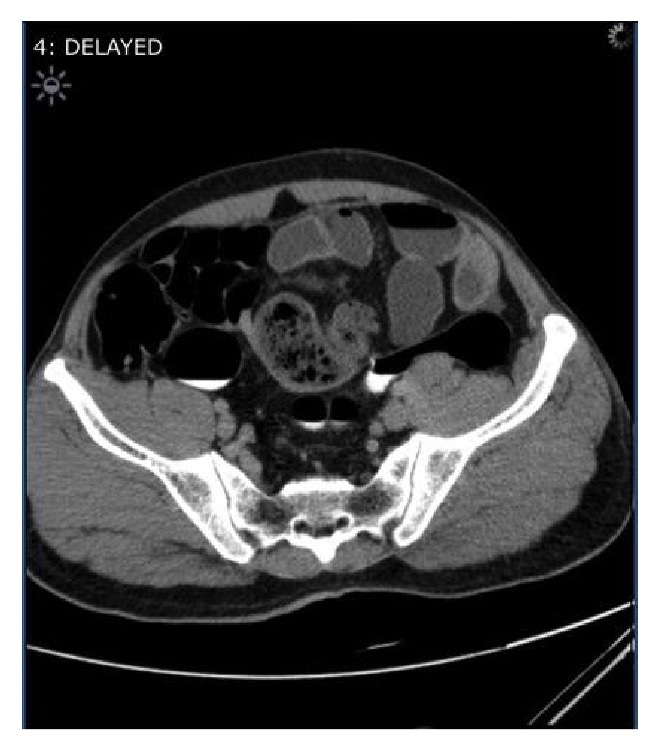


**Figure 2:**
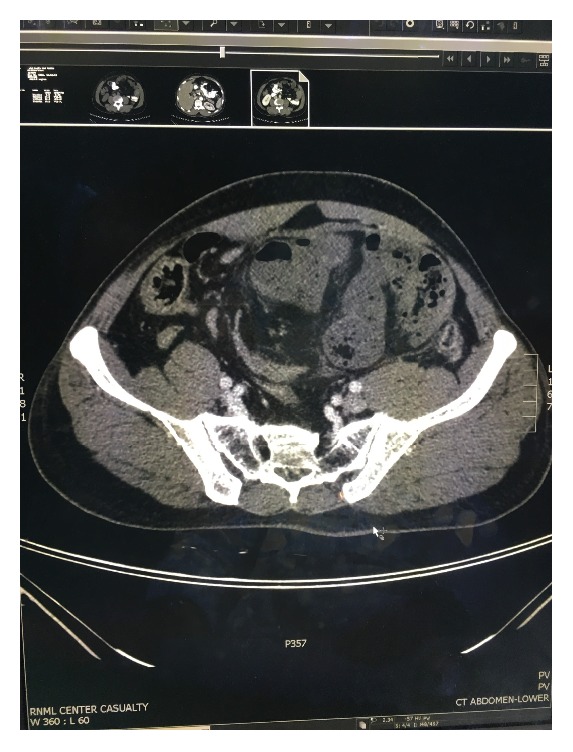


**Figure 3:**
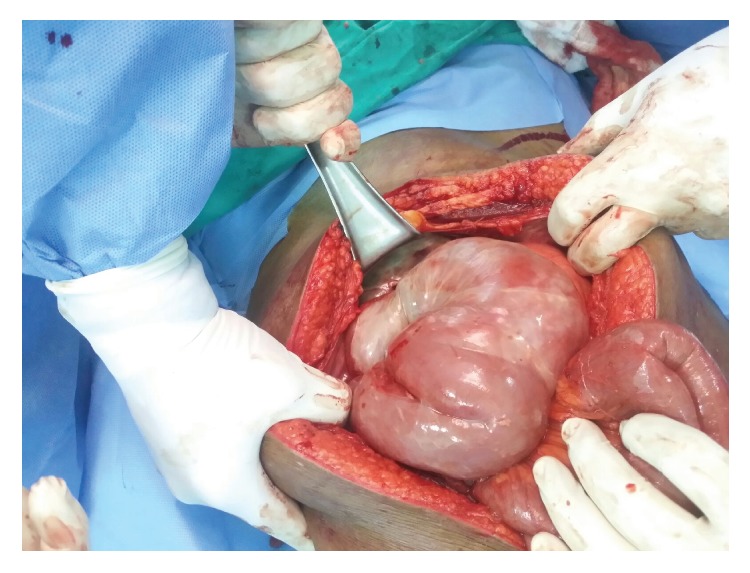


A 50-year-old Pakistani gentleman, who was previously healthy, was admitted to our surgical department with generalized abdominal pain, absolute constipation, and abdominal distention. He had multiple admissions during the previous eighteen months with similar episodes, which were diagnosed as small bowel obstruction, by the clinical picture, and multiple air-fluid levels on abdominal X-rays, and always resolved on conservative measures. He was afebrile and hemodynamically stable. His abdomen was distended with no scars. He had generalized abdominal tenderness and exaggerated bowel sounds. His rectum had remnant of stools on PR examination. He had neutrophilic leukocytosis, but the rest of his labs were within normal limits. His axial abdominal X-ray showed multiple air-fluid levels. A computed tomographic scan of his abdomen, which was performed on the same day of admission, showed evidence of multiple bowel loops dilated in the left side of the abdomen with distal collapse of bowel (transition zone) with swirling sign of the vessels. Additionally, an encasement of the small bowel within a membrane-like sac was observed, with a few calcifications (Figures 1 and 2). No mesenteric thickening, ascitic fluid, or lymphadenopathy was seen. The patient was kept nil per oral, on intravenous fluids and parenteral antibiotics, and nasogastric tube was inserted. The next day the patient became toxic with temperature of 39 degrees Celsius, and the decision was made to take the patient for diagnostic laparoscopy (DL). DL showed amalgamated bowel, encased in a thick fibrous tissue, and then was converted to laparotomy. The fibrous case (Figure 3) was opened, dissected, and separated from the bowel loops. A 30 cm portion of gangrenous ileum, approximately 30 cm away from the ileocecal valve, was identified (Figure 4). The proximal portion of the obstruction bowel was massively dilated. The gangrenous bowel was resected and primary side-to-side anastomosis was performed using GI stapler. There was no lymph node involvement observed. No antiadhesion agent was administered, and the patient's abdomen was closed, and a drain was deployed. The postoperative course was uncomplicated. The drain was removed when it gradually became nil. The patient started passing gas on the eighth postoperative day and passed motion on the tenth postoperative day, and eventually the nasogastric tube was removed. The abdominal distention was gradually decreasing on the right side but remained to have a bulge on the left side. Histopathology of the bowel adherent nodule was consistent with reactive fibrosis with hyalinization and calcifications. The dissected small bowel showed transmural ischemic necrosis, consistent with gangrenous small bowel with free surgical margins. The patient was diagnosed as a case of idiopathic sclerosing encapsulating peritonitis, as his past medical history is unremarkable. The patient was ambulating, was started on clear fluids on postoperative day 10 and soft diet two days later, and was tolerating well. The patient was discharged on the sixteenth postoperative day, with scheduled outpatient clinic visit, but he was lost to follow-up.

## 3. Discussion

Sclerosing encapsulating peritonitis, also known as cocoon syndrome, is a poorly understood and rarely described condition. The pathogenesis is thought to be by the release of fibrin-like material by fibrinogenic cytokines [3]. It is subdivided into idiopathic and secondary types, depending on the cause [4–11]. The cause of the idiopathic type is unknown, hence the name. It is, however, more prevalent in young women living in tropical and subtropical regions but may also be seen in children living in temperate areas or older individuals [12]. In women, retrograde menstruation and retrograde extension of pelvic infections have been thought to be a probable cause of the disease [4, 5]. Peritoneal dialysis is the most common described cause of secondary SEP worldwide [4]. In the literature, most papers about SEP describe its association to the peritoneal dialysis. Rarer causes include SLE, FMF, fibrogenic foreign body, beta-blocker use, ventriculoperitoneal and peritoneovenous shunts, orthotopic liver transplantation, and recurrent peritonitis [7–9, 11]. Our patient, who did not have any significant past medical or surgical history, was considered to be as a case of idiopathic origin.

The clinical presentation of patients with SEP is usually vague, with nonspecific abdominal symptoms, including bloating, nausea, abdominal discomfort, constipation, or vomiting [4, 12], which is why most cases go undiagnosed for a long time. Other patients, such as our patient, present with acute recurrent episodes of intestinal obstruction, which may or may not resolve with conservative management. In the literature, two cases of perforated bowel secondary to SEP have been reported [12].

The preoperative diagnosis of SEP is usually challenging. It takes high experience and knowledge of the disease to suspect its presence. To properly diagnose SEP preoperatively, imaging studies are of crucial importance. These include erect abdominal X-ray films, barium passage radiography, ultrasonography (USG), and computed tomography (CT). Abdominal X-rays would show air-fluid levels and dilated bowel loops in a patient with symptoms of intestinal obstruction. A barium swallow would show an accordion pattern and cauliflower appearance [4, 9, 12]. Abdominal USG would demonstrate dilated small bowel loops encapsulated by a thick, hypoechoic, membrane [4, 12]. CT scan is considered the most useful tool for diagnosing SEP, especially multidetector CT with excellent image quality on coronal, sagittal, and axial planes. The characteristic CT sign is the appearance of loops of small intestine that conglomerate at midline and are encased by a dense mantle without peripheral contrast uptake [4]. Additional findings may include peritoneal thickening, ascitis, intestinal obstruction, calcification of bowel wall, or lymphadenopathy [2]. With these diagnostic tools, the diagnosis of SEP remains challenging given the low index of suspicious, the unawareness about the disease process, and the unavailability of high quality CT machines in some centres. SEP in most patients is eventually diagnosed by intraoperative findings and histopathological studies [7]. Characteristic histopathological features of biopsied peritoneal encasement may include fibroconnective tissue proliferation, inflammatory infiltration, and dilated lymphatic vessels. Although these are not pathognomonic to SEP, they usually support the diagnosis [2].

The management of SEP depends on the presentation of the patient. In asymptomatic patients with idiopathic SEP, regular follow-up is all that is required [9]. Patients with mildly symptomatic cases are suitable for conservative management. These with signs and symptoms of uncomplicated intestinal obstruction can be managed conservatively with intestinal rest, nasogastric decompression, and nutritional support [1, 5]. Patients in which the symptoms do not resolve may be treated with anti-inflammatory and antifibrinogenic drugs such as tamoxifen, steroids, colchicine, azathioprine, and mycophenolate [1]. However, these treatments have only been reported to be used in secondary types of SEP with no evidence of their use in primary type. In the last category of patients, who present with intestinal obstruction complicated by unresolution of the obstruction by conservative measures, perforation, or ischemia, surgical intervention may be inevitable. Different surgical options include adhesiolysis with excision of the membrane and resection and anastomosis [13]. Resection should be avoided except in clearly indicated patients, such as those with bowel ischemia, as it may lead to more complications, such as anastomosis leak and short bowel syndrome, which will increase the morbidity and mortality of the condition [5, 9]. The role of laparoscopy in the management of SEP remains unclear. There is limited evidence in the literature for a successful laparoscopic membrane excision and adhesiolysis. The advantage of laparoscopy is that it can be added as a tool that aids in diagnosis of the condition [10]. The use of antiadhesion measures intraoperatively has no clear evidence whether or not it may prevent the recurrence of the condition.

In conclusion, SEP is a rare, poorly understood condition in which a fibrous membrane encapsulates the bowel, possibly leading to intestinal obstruction and its consequences. Preoperative diagnosis is usually challenging. The use of the CT scans along with other imaging techniques may assist in reaching a correct diagnosis and subsequently the proper management. Although conservative management remains preferred in mildly symptomatic patients, surgical intervention is often required to prevent or deal with the complications of intestinal obstruction. A minimally invasive approach should be attempted to avoid troublesome complications.

## Figures and Tables

**Figure 4:**